# Analysis of Cattle Social Transitional Behaviour: Attraction and Repulsion

**DOI:** 10.3390/s20185340

**Published:** 2020-09-18

**Authors:** Haocheng Xu, Shenghong Li, Caroline Lee, Wei Ni, David Abbott, Mark Johnson, Jim M. Lea, Jinhong Yuan, Dana L. M. Campbell

**Affiliations:** 1 School of Electrical Engineering and Telecommunications, University of New South Wales, High St, Kensington, NSW 2052, Australia; xhc122394@gmail.com (H.X.); j.yuan@unsw.edu.au (J.Y.); 2 Data61, Commonwealth Scientific and Industrial Research Organisation (CSIRO), Marsfield, NSW 2122, Australia; wei.ni@csiro.au (W.N.); david.a.abbott@csiro.au (D.A.); mark.johnson@csiro.au (M.J.); 3 Agriculture and Food, Commonwealth Scientific and Industrial Research Organisation (CSIRO), Armidale, NSW 2350, Australia; caroline.lee@csiro.au (C.L.); jim.lea@csiro.au (J.M.L.); dana.campbell@csiro.au (D.L.M.C.)

**Keywords:** animal behaviour, social behaviour, leader animals, unsupervised learning, multidimensional scaling (MDS), agglomerative hierarchical clustering (AHC), K-means clustering

## Abstract

Understanding social interactions in livestock groups could improve management practices, but this can be difficult and time-consuming using traditional methods of live observations and video recordings. Sensor technologies and machine learning techniques could provide insight not previously possible. In this study, based on the animals’ location information acquired by a new cooperative wireless localisation system, unsupervised machine learning approaches were performed to identify the social structure of a small group of cattle yearlings (n=10) and the social behaviour of an individual. The paper first defined the affinity between an animal pair based on the ranks of their distance. Unsupervised clustering algorithms were then performed, including K-means clustering and agglomerative hierarchical clustering. In particular, K-means clustering was applied based on logical and physical distance. By comparing the clustering result based on logical distance and physical distance, the leader animals and the influence of an individual in a herd of cattle were identified, which provides valuable information for studying the behaviour of animal herds. Improvements in device robustness and replication of this work would confirm the practical application of this technology and analysis methodologies.

## 1. Introduction

Studying individual interactions in animal groups improves our understanding of social dynamics, networks, and hierarchies. The outcomes of such research applied to livestock groups can potentially improve livestock productivity, enhance management practices, and provide scientifically-validated recommendations for regulations and legislation of the farming industry [1]. Cattle are typically social animals, living within groups in either intensive or pasture-based farming systems. Social relationships between individual cattle have been reported [2,3,4] as well as differences in dominance status [5,6,7]. Leaders and followers have been identified during grazing movement patterns within groups of cattle where age, dominance, and social position can affect which individuals are the most influential [6]. While social networks can have positive benefits to the group through affiliative bonds, they can also create social tension which can result in aggression and competition [2]. Competitive behaviours can impact resource access in group settings, which may have negative implications for production animals [8]. The ability to measure social relationships will enable a greater understanding of how individuals within a group influence each other, which may inform improvements in management practices, housing design, and resource provision.

Behavioural analysis of animals is non-trivial. Accurately observing animal behaviour requires minimal human interference, and reliance on visual observations alone can be subjective and unreliable [9]. The feasibility of live observations and time-consuming processing of video recordings can limit the continuity of data able to be collected. The development of automated data collection using devices that are attached to animals has enabled greater insight into both individual and group behaviours not previously possible, although they cannot reliably detect all behaviours that a human-observer can [10]. Many studies have validated the application and value of on-animal accelerometer devices that can automatically quantify exhibited behaviours of cattle such as lying, grazing, ruminating and walking [11,12]. Robust devices are available commercially with the potential to monitor animals in research settings and on-farm to detect changes in typical behavioural time budgets that could be indicative of underlying problems such as poor health [13]. Automated devices that can reliably quantify animal positioning patterns relative to their conspecifics represent an avenue for understanding beyond the individual to quantify social relationships, interactions between animals, group cohesion and identify leaders that may influence group behaviour.

Social networks and/or movement patterns in cattle have previously been determined using a range of methodologies, including live observations of proximity, leader animals, and scoring of social rank [4,6,14], GPS data [7,15], image analysis of proximity based on unmanned aerial vehicle (UAV) footage [16], and collar devices that register at antennas [3,17]. On-animal contact or proximity loggers using ultra-high frequency signals are a technology that researchers are increasingly using to monitor social interactions and networks in cattle. This facilitates understanding of group affiliative or avoidance relationships [18], male–female interactions to detect oestrous [19], associations between maternal and pregnant cows [20], and cow–calf interactions [21]. However, the data from proximity loggers can be prone to error [22], signals can be interfered with by metal or other animals, and they do not provide exact geo-spatial animal positioning. The spatial movement patterns and interactions of animals measured by precise positioning systems with a high update rate present opportunities to quantify animal behaviour automatically and more accurately [23,24], and provide insights that cannot be obtained from visual classification alone [3,17].

Data generated from both proximity, GPS, and localisation devices can total thousands of registered datapoints and traditional methods of analysis may be insufficient for revealing potential patterns of group behaviour. Machine learning, in particular, unsupervised learning or clustering, has been applied to process the location data of animals, to study survival status, living habits, migration range and path of animals, to obtain knowledge of animal behaviour and social interactions [25,26]. ‘Big data’ techniques also have many potential applications for improvements in livestock husbandry systems as detailed in several reviews [27,28]. Machine learning algorithms such as stratified cross validation (SCV) approaches, binary trees, linear discriminant analysis classifiers, and decision trees have been applied to data from on-animal devices to reliably classify different behavioural patterns such as chewing variation or types of activity (e.g., [29,30,31,32]), or to GPS data to detect grazing, resting, and walking [33]. Machine learning has also been applied to determine if changes in behavioural patterns as detected by on-animal sensors could predict life events such as calving [34] or health problems such as lameness [35]. Physiologically, machine learning has been applied to detect estrous via vaginal sensors [36] and can also be used for management decisions such as the prediction of insemination outcomes [37]. Machine learning techniques are applied to positional data which could also provide new insights into the behaviour of herds. For example, K-means clustering has been applied to classify cows into active and inactive groups [38]. Hierarchical clustering was applied to the dissimilarity produced by Dynamic Time Warping to analyse the moving patterns of hens [39] and the ‘routine’ behaviour patterns of free-range laying hens were identified with clustering algorithms [40]. However, none of these studies have been able to analyse the interactions in a dynamic and unstable state.

Although a local-position measurement system and positional loggers have been used for some research [3,17], in most studies, the geo-spatial locations of individual animals were obtained by the GPS system, which may not provide enough information about the social behaviour of livestock due to the low positioning accuracy (typically 2–5 m). Other systems [17] can provide higher accuracy than GPS but have a limited operating range. Furthermore, proximity loggers (e.g., [22]) cannot quantify the closeness between animals because they only provide binary information as to whether two animals are within a predefined distance of each other.

Thus, the purpose of this research was to develop an efficient and accurate approach to study the social behaviour of cattle groups based on the novel application of a high-accuracy wireless tracking system and the latest machine learning technologies. Novel data analysis algorithms were developed and applied to the experimental data, which enabled the parameterising and visualising of the bonding between different animals, the influence of individual animals, and the strong repulsion (or avoidance) between individuals.

## 2. Experimental Design and Data Capture

### 2.1. Ethical Statement

The experiment was approved by the CSIRO FD McMaster Laboratory Chiswick Animal Ethics Committee prior to the start of the experimental period (ARA 18-06).

### 2.2. Animals and Experimental Protocol

**Figure 1:**
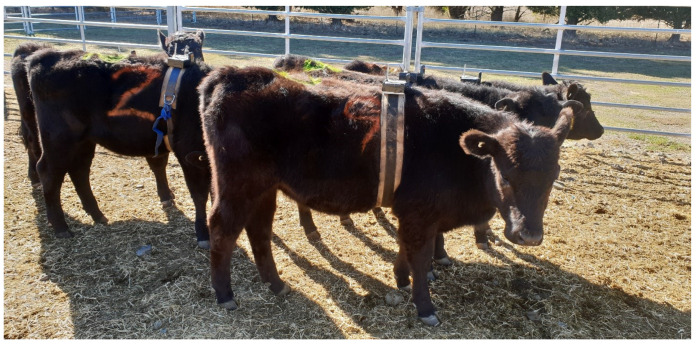
Some of the cattle fitted with localisation devices on a girth strap including spray-painted individual identification numbers.

**Figure 2:**
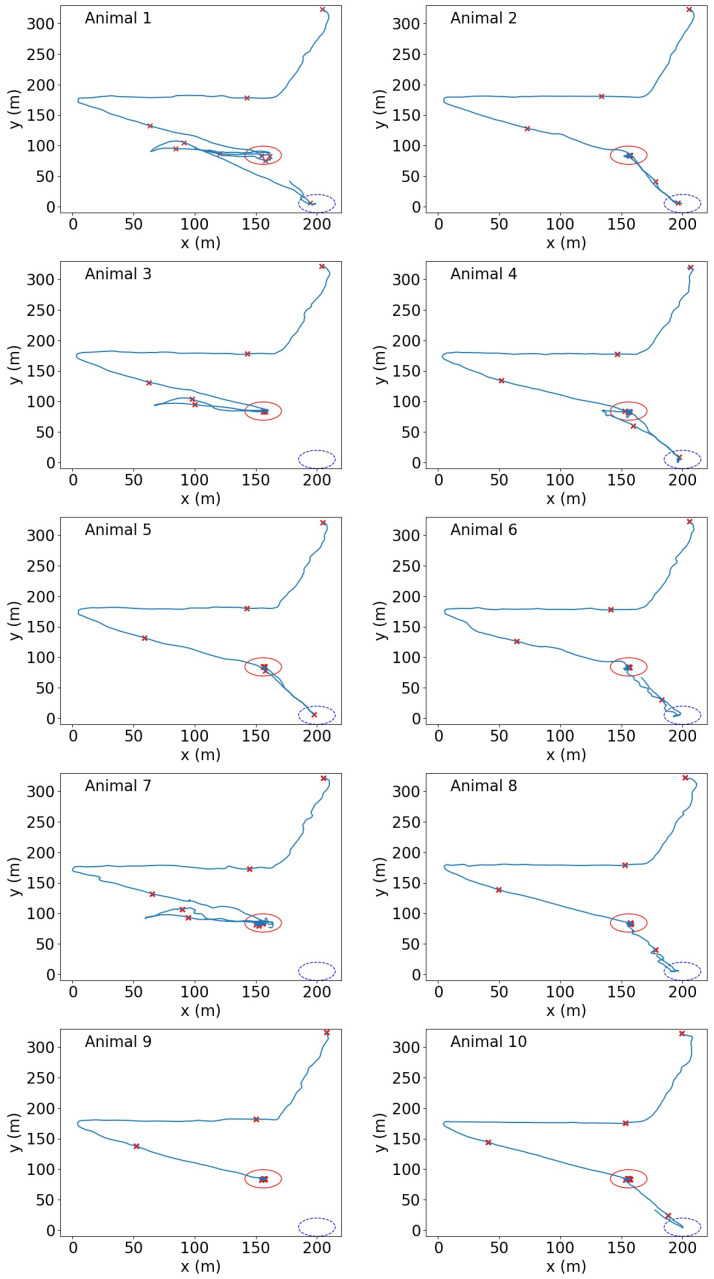
The trajectory of each animal acquired by cooperative wireless localisation across the 25-min trial period. All trajectories commenced in the upper right corner as animals left the yards and were led into the test paddock by a handler. The red circle and blue circle indicate the locations of feed trough and water trough, respectively. The red “×” marks highlight the animal positions at a number of time instances that are 150 s apart during the experiment.

The experiment took place at the Chiswick research site (Armidale, NSW, Australia) of the Commonwealth Scientific and Industrial Research Organisation (CSIRO) in August 2018 (late autumn, clear skies with no rain, maximum $18{\;}^{{}^{\circ}}$C) in a rectangular-shaped grassed test paddock (150×50 m). A single group of three Angus steers and seven Angus heifers (approximately 12–18 months old, mean body weight ± SE 261.2 ± 10.1 kg, in visibly healthy condition), were painted with an identification number on each flank using livestock tail paint (Leader Products Pty Ltd., Craigieburn, VIC, Australia) and fitted (in a crush) with a wireless device that consisted of a GPS receiver module for coarse localisation (and also for benchmarking purposes) and a custom-designed wireless transceiver for fine-grained localisation and tracking. The wireless tracking assemblies were attached to a girth strap (using cable ties) that was fitted to the animal just behind the forequarter (Figure 1). The animals were socially acclimatised to each other as they had been living together in the test paddock for 10 weeks prior to the trial (including two additional animals not used in the trial due to device availability). On Day 1, the animals were fitted with the devices and placed into the test paddock for approximately four hours to validate that the system was functioning correctly and allow acclimation to the devices. A water trough was present in the paddock. The devices were removed to be charged overnight and the animals were presented with a concrete trough of feed in the yards to become acclimated to obtaining feed in a competitive feeding set-up. The small concrete trough (535 mm diameter 295 mm height) restricted access to feed (lucerne hay on top, oaten chaff mixture inside) to 3 to 4 animals at a time. On Day 2, the animals were again led into the test paddock with access to the feed trough containing approximately 5 kg of the chaff mixture. The animals approached and started feeding and jostling for access to feed almost immediately. Positions were recorded on the devices during two competitive feeding situations for approximately 25 min each to validate device functionality and recording in this scenario. Animals were brought back for device removal and battery charging overnight. On Day 3, the animals were refitted and led from the yards into the paddock by a handler carrying hay for a final competitive feeding scenario which comprised the data analysed in this study. The animals walked away from the feed once it was all consumed. The data were recorded for 25 min starting from when all animals left the yards and were walked down into the paddock (see Figure 2 for animal movement trajectories).

### 2.3. The Wireless Tracking Platform

The instantaneous location of each animal was captured with a custom-designed localisation platform which provided a positioning accuracy and coverage that significantly outperforms commercially available systems. The fitted device (Figure 1) contained a GPS module and a ‘wireless ad-hoc system for positioning’ (WASP) node [41] developed by the CSIRO. The WASP system operated in the 5.8 GHz ISM band, utilising a bandwidth of 125 MHz for range measurements based on time-of-arrival (TOA). Each unit was 12 cm × 8 cm × 4 cm in dimension and weighed about 400 g. The devices formed a multi-hop ad-hoc network during operation, allowing all the available ranging information between pairs of nodes to be collected. Detailed information about the WASP system can be found in [41] and references therein. The locations of animals were estimated offline by fusing GPS position measurements and the relative range measurements obtained by the WASP system with an Extended Kalman Filter (EKF) [42]. The details are provided in Appendix A . The system can locate the animals to an accuracy of 15 cm, which provides precise spatio-geometry information about the group that cannot be obtained otherwise, e.g., by using GPS alone.

## 3. Data Analyses

The data used for analyses were recorded during one 25-min competitive feeding scenario with the 10 animals during the morning. To characterise the closeness between pairs of animals, a logical distance was defined based on the ranking of physical distances. Two clustering algorithms were then performed to study the social behaviour of animals: K-means clustering based on dimension reduction and agglomerative hierarchical clustering. K-means clustering was also applied based on the physical locations of animals, where each group contained the animals that were physically close to each other.

### 3.1. Graph-Theoretic Interpretation of Intra-Group Closeness

To measure the (logical and statistical) closeness between any pair of animals in the experimental group, the ranks of their distance were used with reference to their respective distances to the rest of the group. At every time instance, the ranks were recorded, with these ranks indicating how closely two animals preferred to stay together and interact throughout the experimental period. Because the animals could roam throughout the test paddock, the physical distances could vary drastically, rendering them less meaningful and potentially biased or distorted, thus ranks were used.

Using the distance ranking of every animal with respect to every other animal in the experimental group, a normalised average distance ranking of any animal *i* was evaluated with respect to animal *j* (i≠j) to measure the closeness of animal *i* to animal *j*, as given by

(1) $$W_{ij}\;=\;\sum_{t=1}^{T}\frac{R_{ij}\left(t\right)}{T},$$

where $R_{i,j}\left(t\right)$ denotes the ranking of the distance of animal *i* with respect to *j* (i≠j and 1≤i,j≤N) at timeslot *t*. The total number of animals in the group was N = 10, so $1\leq W_{i,j}\leq 10$. Here, $W_{i,j}$ was not necessarily equal to $W_{j,i}$ (i≠j). This was because two animals could have different social attachments towards each other and one of them may value the bond more than the other. An N-dimensional square matrix W was then constructed from $W_{i,j}$, which could be graph-theoretically interpreted as a bidirectional, weighted graph representing the overall closeness between pairs of animals.

### 3.2. K-Means Clustering Based on Dimension Reduction

Multidimensional scaling (MDS) is an effective means of visualising the level of similarity between samples in a dataset [43]. It has been widely used to translate the pairwise “distances” among a set of samples into a constellation of points mapped into an abstract Cartesian space [44]. MDS was applied to transform the N × N matrix W into an N×2-dimensional matrix denoted by X, each row of which represented the position of an animal in an abstract 2-D space, which could be used to cluster the animals and reveal their social bond. To the best of the author’s knowledge, this clustering over a bidirectional and (differently) weighted graph has not yet been published in the literature.

A symmetric matrix D was first constructed from W, with $D_{i,j}\;=\;W_{i,j}\times W_{j,i}$. Then, a matrix B was defined as follows [43]

(2) $$B\;=\;-\frac{1}{2}CD_{2}C,$$

where $D_{2}\left(i,j\right)\;=\;D_{i,j}^{2}$ was the square of dissimilarity (distance) of animal *i* with respect to *j*, $C\;=\;I_{N}\;-\;\frac{1}{N}J_{N,1}J_{1,N}$, $I_{N}$ was an N×N identity matrix, and $J_{N,1}$ was the N×1 all-one vector. By taking the eigenvalue decomposition of B, i.e., $B\;=\;V\Lambda V^{†}$, a 2D representation could be made of the edge weight matrix as given by

(3) $$X\;=\;\Lambda_{2}^{\frac{1}{2}}V_{2}^{†},$$

where $\Lambda_{2}$ was a diagonal matrix constructed from the two largest eigenvalues in Λ, $V_{2}$ were the corresponding eigen vectors in the columns of V, the superscript ${\left(\cdot \right)}^{†}$ stood for transpose.

Inspection of X in the 2D abstract Cartesian space identified the behavioural similarities (closeness) between the animals. The K-means clustering algorithm was first applied [45] to reveal the animals that had a stronger bond with each other than with the rest of the group. The number of clusters was specified by K, also referred to as a “K-value” [46]. Given the *K*-value, K-means clustering randomly selects K points as the initial centroids and evaluates the distance from every sample to the K centroids one after another. Then, the centroids of the clusters are re-evaluated, and the clusters of the rest of the samples are updated accordingly [47]. This repeats until the clusters stop changing. During the analysis, the number of clusters K was chosen automatically with silhouette analysis.

The variation of the distance ranking of the animals throughout the experimental period was also further analysed to understand repulsion between some individuals. A new asymmetric square matrix, $D_{v}$, capturing the variation between any pair of animals was given by

(4) $$D_{v}\left(i,j\right)=\frac{1}{T}\sum_{t=1}^{T}{\left(\frac{1}{R_{i,j}\left(t\right)}-\frac{1}{T}\sum_{t=1}^{T}\frac{1}{R_{i,j}\left(t\right)}\right)}^{2},$$

where $R_{i,j}\left(t\right)$ was the ranking animal *i* with respect to *j* at timeslot *t*, as defined earlier. The matrix indicated the amount of variation or dispersion between the connection of animal pairs. Following the method described in Section 3.2, $D_{v}\left(i,j\right)$ was converted into a symmetric matrix based on $S_{i,j}\;=\;D_{i,j}\;\times \;D_{j,i}$ and then MDS and K-means clustering were applied to study the repulsions.

### 3.3. Agglomerative Hierarchical Clustering

Agglomerative Hierarchical Clustering (AHC) is a bottom-up clustering method that can classify the samples based on pair-wise distances (or also known as “affinity”) [48]. At the beginning of the AHC operations, each sample is considered as a single cluster. Then the two closest clusters are selected to merge in each iteration until only a single cluster remains (referred to as the “root node”). Finally, AHC can establish a dendrogram which is a one-dimensional tree to display the closeness (or similarity) of an individual to its closet (or most similar) cluster [49].

To apply the AHC algorithm, the affinities between the animals were first defined. This started by defining the outdegree which characterised the correlation from an animal to a cluster, and the indegree of an animal from a cluster that reflected the density near the animal [50].

For any animal *i*, the average indegree from cluster C and the average outdegree to the cluster were defined as

(5) $$\begin{array}{cc} deg_{i}^{-}\left(C\right) & =\frac{1}{\left|C\right|}\sum_{j\in C}W_{j,i}\\ deg_{i}^{+}\left(C\right) & =\frac{1}{\left|C\right|}\sum_{j\in C}W_{i,j} \end{array}$$

where $deg_{i}^{+}\left(C\right)$ stood for the indegree from C to animal *i*, and $deg_{i}^{-}\left(C\right)$ stood for the outdegree from an animal to the cluster *C*. |C| was the size of C. and $W_{j,i}$ was the weighting coefficient of *j* and *i* defined earlier. If cluster C contained animal *i*, both the indegree and outdegree were expected to be large. Therefore, the affinity between cluster C and animal *i* could be measured by the product of the indegree and outdegree [50]:

(6) $$A_{i\to C}=deg_{i}^{-}\left(C\right)\times deg_{i}^{+}\left(C\right).$$

Likewise, the affinity between two clusters $C_{a}$ and $C_{b}$ (a≠b) could be given by

(7) $$A_{C_{b}\to C_{a}}=\sum_{j\in C_{b}}deg_{i}^{-}\left(C_{a}\right)\times deg_{i}^{+}\left(C_{a}\right)$$

Based on the weighting coefficients in Table 1, a symmetric affinity between clusters $C_{a}$ and $C_{b}$ could be written as

(8) $$A_{C_{a},C_{b}}\;=\;A_{C_{b}\to C_{a}}+A_{C_{a}\to C_{b}}.$$

At the beginning of AHC, every animal formed a cluster of its own, and the indegree and outdegree of the cluster were $deg_{i}^{-}=W_{j,i}$ and $deg_{i}^{+}=W_{i,j}$, respectively. Based on (8), the affinity between two clusters was $A_{i,j}\;=\;A_{i\to j}+A_{j\to i}=W_{i,j}\times W_{j,i}$. Then, clusters started to merge with each other based on the maximum affinity until only a single cluster remained [51], as described earlier.

### 3.4. Herd Behaviour Based on Geo-Spatial Locations

In addition to the logical distances defined in Section 3.1, the K-means clustering technique was also performed based on the physical locations of animals, and each group contained the animals physically close to each other. The clustering algorithm was performed on the positional data at a number of time instances that were 1500 timeslots (150 s) apart, as indicated in Figure 2. The statistics on the relative positions between animals (e.g., an animal stays close to others, multiple animals stay close together) were studied, which provided information on the connections and social status of the animals.

## 4. Results

### 4.1. Captured Positional Data

The location estimations were generated at a fixed rate of 10 Hz during the experiments, which included the x- and y-coordinates and the associated timestamp. A total of 17,933 valid position records were obtained for each animal during one 25-min competitive feeding scenario. Figure 2 shows the trajectory of each animal during the period.

### 4.2. Graph-Theoretic Interpretation of Intra-Group Closeness

**Figure 3:**
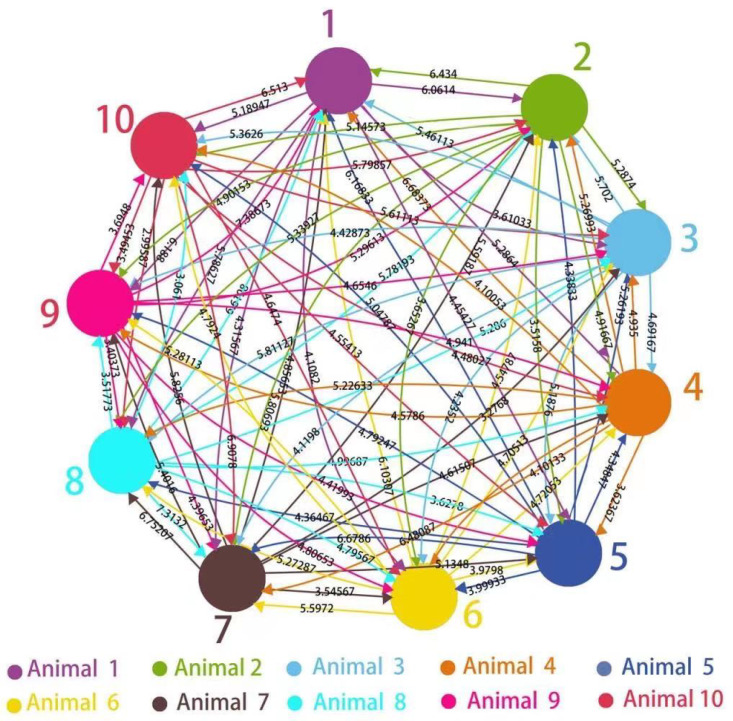
The new graph-theoretic interpretation of the closeness of the group of animals in the experiment, where each vertex corresponds to an animal and each directional edge is associated with a weight $W_{i,j}$ to measure the time-averaged closeness of animal *i* to animal *j* against the rest of the group, where $1\leq W_{i,j}\leq 10$.

Table 1 shows the closeness between every pair of animals in the experiment as characterised by $w_{i,j}$ defined in Section 3.1. Each row represents the degree of attention that one animal attracted from each of the other individuals. Each column shows the degree of attention from the other animals to the animal. The values in the table can be interpreted as the weights of the directional edges in a bidirectional, weighted graph, where the vertices stand for individual animals and the edges indicate the closeness of different pairs of the animals as plotted in Figure 3.

### 4.3. K-Means Clustering Based on Dimension Reduction

**Figure 4:**
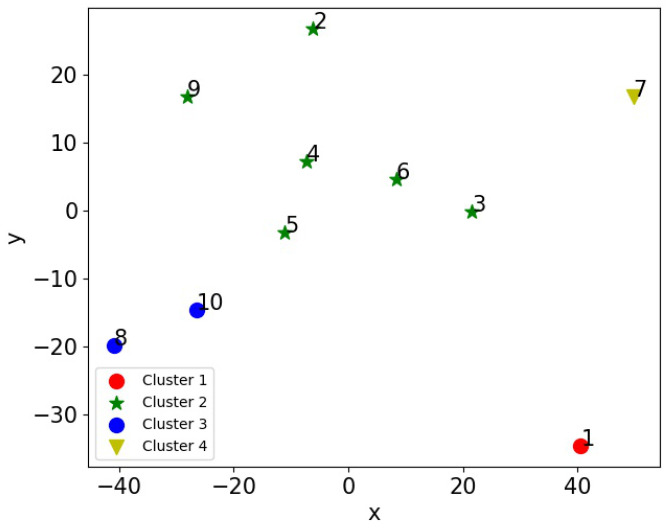
The locations of 10 cattle in an abstract 2-D space obtained by MDS based on the pairwise affinities. Note that the coordinates on the *x* and *y* axes have no physical meaning but indicate the relative closeness between animals. Animals closer to each other in the plot have stronger interactions. The cattle are grouped into four clusters by the *K*-means clustering algorithms, as indicated by the four different markers.

Figure 4 illustrates the relative closeness between animals in an abstract 2D space obtained by MDS, along with the result of K-Means clustering. It is shown that animal 6 was at the centre of the group, indicating it was the statistically closest animal to the rest of the group throughout the experiment. In other words, animal 6 was the most influential individual of the group, with the majority of the rest of the group preferring to be around it and follow its movements, for example, animals 2, 3, 4, 5, and 9. In contrast, a few animals, i.e., animals 1 and 7, displayed strong separation. Animals 8 and 10 were grouped into one cluster; animals 2 to 6 and 9 formed another cluster; and animals 1 and 7 were two separate clusters.

**Figure 5:**
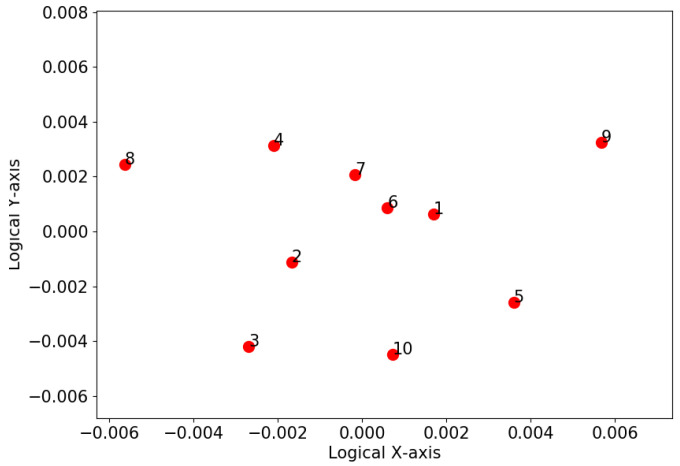
Mapping the social interaction variation of each animal pair by using multidimensional scaling. Note that the coordinates on the x and y axis have no physical meaning but indicate the degree of variation for the closeness between animals. Animals close to each other in the plot show a high degree of variation in their social interaction.

Figure 5 shows the result of mapping the deviation matrix (i.e., $D_{v}$ in (4)) to a two-dimensional space by utilising MDS, where the x and y-axes indicate the logical coordinates of each animal. This shows that animals 1 and 7 were both close to animal 6. This indicates that the closeness between them stayed unchanged. The rankings between animals 6 and 2, animals 6 and 5, and animals 6 and 4 experienced a medium level of variation. By comparing it with Figure 4, where animals 2, 4, 5, and 6 were logically close to each other and grouped into the same cluster, we presume that animals 2, 4, 5, and 6 could have a similar level of social status.

### 4.4. Agglomerative Hierarchical Clustering

**Figure 6:**
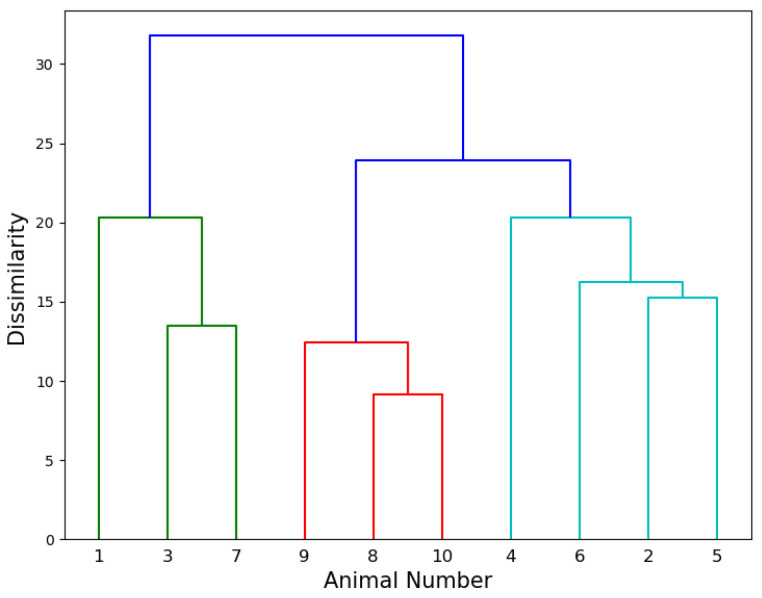
The result of agglomerative hierarchical clustering based on the affinity between animals. The height of the fusion on the y-axis indicates the dissimilarity between two animals/groups. Animals/groups are further away from each other when the height of the fusion is greater.

Figure 6 shows the resulting dendrogram of AHC of the experimental data, where the x-axis indicates the number of animals, and the y-axis quantifies the dissimilarity between different clusters. This figure shows that animals 8, 9 and 10 were “closer” to each other than the rest of the group, as were animals 2, 4, 5, and 6. The results are similar to those shown in Figure 4. Compared to the one-dimensional result of AHC, one advantage of using MDS is that the two-dimensional logical coordinates and distances between animals can be visually seen.

### 4.5. Herd Behaviour Based on Geo-Spatial Locations

**Figure 7:**
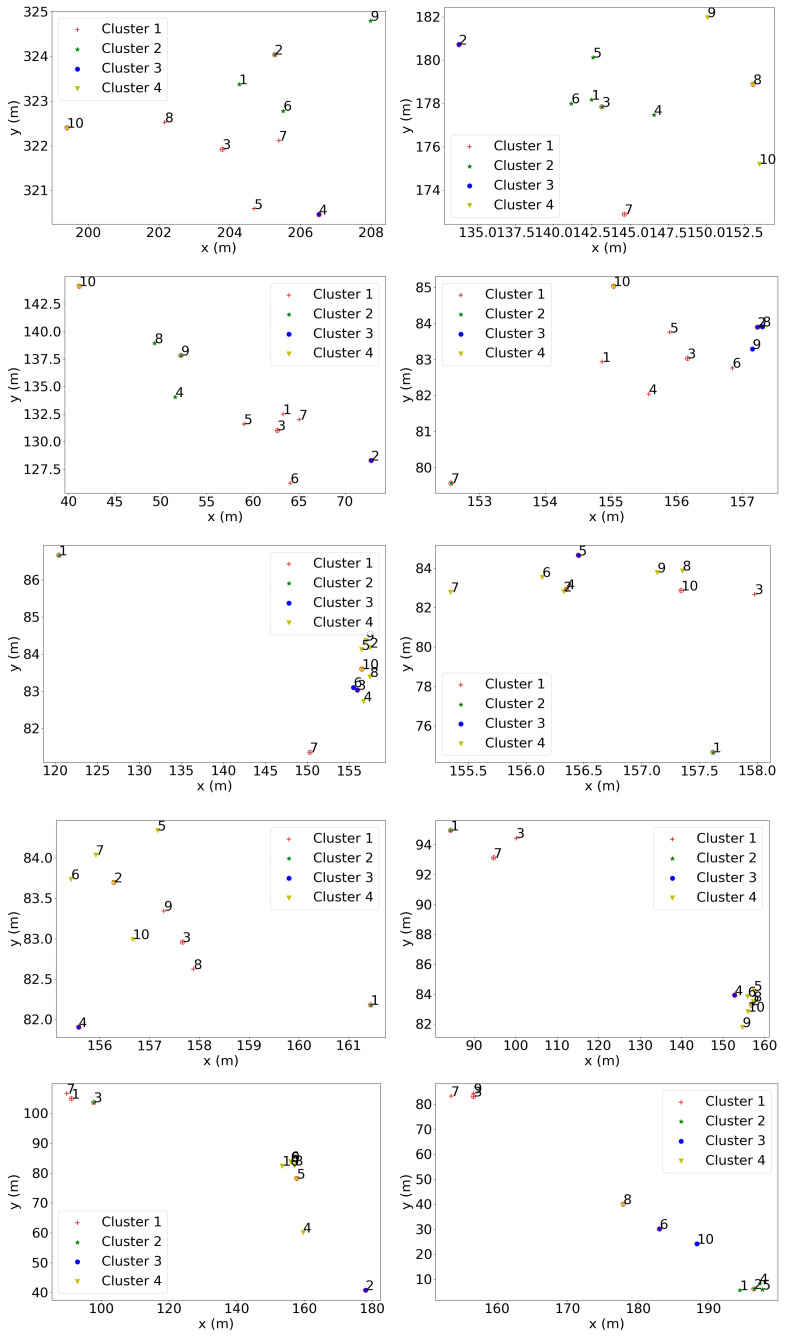
The *K*-means clustering results at a number of time instances that were 150 s apart during the experiment. The x and y-axes indicate the physical coordinates of each animal, and the “∘”, “★”, “▽”, and “+” symbols represent different clusters. The red-circled points are the locations of the animals that were closest to the centroids and were considered the ‘connectors’.

Figure 7 shows the results of physical position-based K-means clustering applied to the snapshots indicated in Figure 2. The number of clusters was 4. The red-circled points are the location of the animals which were closest to the centroids and considered the “connectors”.

**Figure 8:**
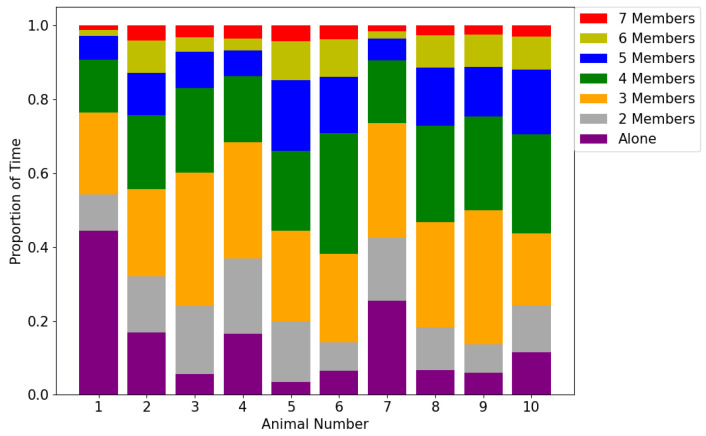
The proportion of time that an animal stayed with other individuals. The x-axis shows the animal number. The proportions of time that an animal was alone or within a cluster which had a different number of members are represented in different colours of the columns.

**Figure 9:**
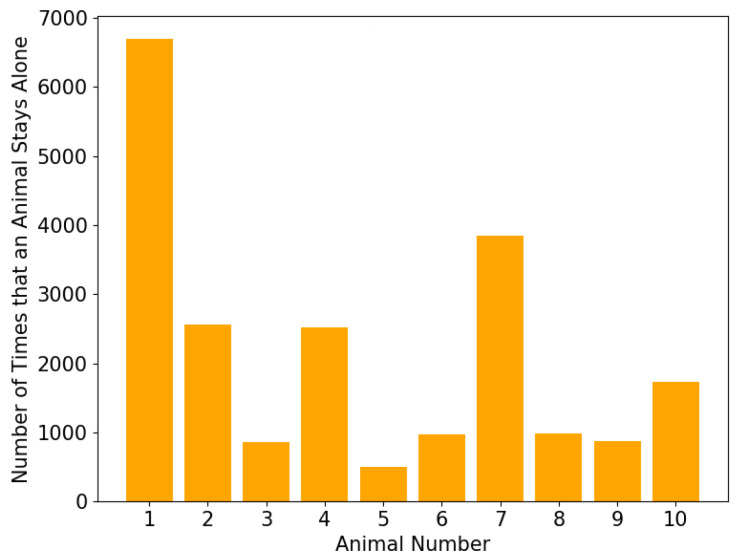
The number of time slots that each animal stayed alone throughout the experimental period of 17,933 time slots.

**Figure 10:**
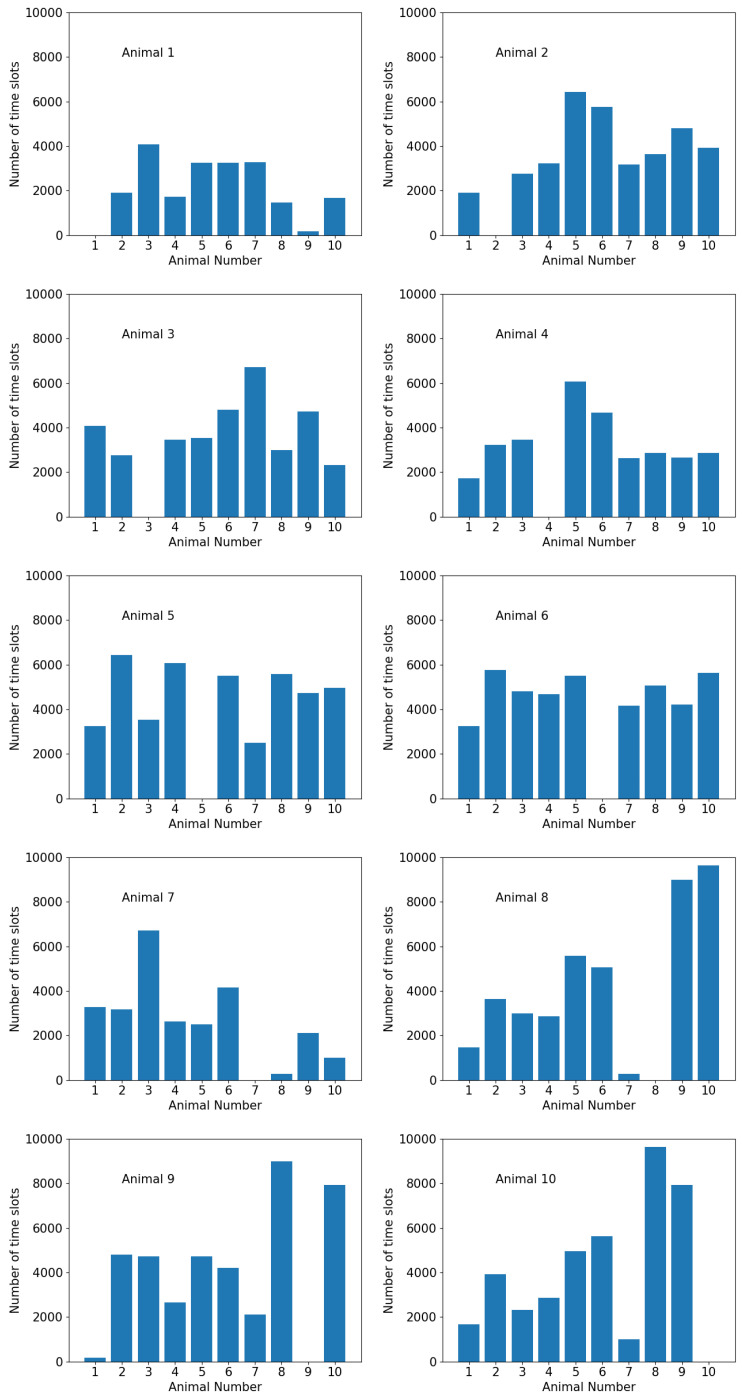
The number of times that each animal was grouped with another as one cluster throughout the experimental period of 17,933 time slots.

The same approach was applied to all timeslots within the captured dataset, and the statistics on the results are shown in Figure 8, Figure 9 and Figure 10.

It can be seen from Figure 8 that the majority of the animals spent most of their time in a cluster with three or four members, but the proportion of time spent alone varied. This can be better observed in Figure 9 which indicates the number of instances that an animal stayed alone (i.e., did not form a cluster with any other animals according to the *K*-means clustering analysis). This shows that animals 1 and 7 spent most of their time alone, totaling 6690 and 3842 timeslots respectively (out of the total 17,933 timeslots). This indicates that animals 1 and 7 were less connected to the others. Figure 10 shows the number of timeslots that an animal stayed with another animal in the same cluster. Animal 6 was most closely related to the other animals, because it had the greatest number of animals that were highly bonded with it. Additionally, animals 1 and 7 had a smaller number of animals that appeared in the same cluster as them over time, indicating they were less connected with the others. The other animals had a similar number of animals that they were connected with, suggesting that they had no significant preference. These outcomes show similar patterns as the results of MDS shown in Figure 4, where animal 6 was in the centre of the abstract 2D space, and animals 1 and 7 were treated as two separate clusters due to their larger distances from the others. It can be presumed that animal 6 was the most influential one, whereas animals 1 and 7 were less connected to the other animals and were highly repelled by animal 6.

## 5. Discussion

This study applied novel wireless localisation devices to measure the geo-spatial locations across time of a group of 10 yearling cattle at fine spatial resolution during walking from the yards into a paddock area where a competitive feeding set-up was placed. The data points collected across the 25-min study period were analysed with two machine learning clustering algorithms both of which demonstrated that one animal was most influential or central within the group, that animals preferred to be in groups of 3 or 4 individuals, and that two individuals spent more time alone and were likely repelled by the central individual. This first application of these devices and analysis methods demonstrate the information that can be gained about social interactions and networks in animal groups from short observation periods.

The wireless tracking system used in the experiment combined a range-based positioning system (WASP) and GPS receivers for improved accuracy. It was robust against measurement errors by exploiting all available measurements, including the pairwise range measurements between animals. To obtain positioning data with the highest quality for analyses, several repeated measurements were conducted on the animals and those with outage or accuracy loss in GPS measurements were excluded. Average spatial accuracy of 15 cm was achieved in these outdoor experiments which is greater than previous reports of 50 cm accuracy in cattle sensor systems [3,17]. However, these systems were used indoors, and the WASP system would have similar accuracy of 50 cm if used inside due to the reflection of radio signals off metal structures. The operation range of the system is mainly determined by that of the WASP devices, which lies between 50 and 100 m in outdoor environments. A WASP device can only be located if it can measure ranges to three or more anchors (which are WASP devices deployed at known locations). However, range measurements between animals can be exploited to locate those that do not have enough anchors in range and improve the positioning accuracy, as was done in the current experiment. The coverage area of the system could be extended arbitrarily with proper deployment of anchors. The system during the trials had a limited battery life of two hours. This time could be extended in future trials by using larger batteries but device size would be dependent on the bodyweight of the animals used to ensure minimal effects on the animal’s behaviour.

Benefitting from the high positioning accuracy, the data analysis performed in the paper was based on the geo-spatial positions of individual animals, which provides more precise information on the interactions between animals than proximity loggers [22]. In addition, a logical distance was introduced to characterise the closeness between animals, which delivers more accurate and robust results than using physical distances [7]. In particular, the relative positions between animals may be scale-invariant, i.e., the geometrical topology of the animal group could enlarge/shrink depending on the animals’ activity while the relative closeness among animals remains unchanged. Using logical distance enables accurate identification of the social structure of an animal group undergoing different activities, such as grazing, resting, and walking to new areas. It is shown that the results obtained with logical distances were similar to those obtained with physical distances in our experiment. Further research could be conducted to validate the advantage of using logical distances on experiments of extended durations.

The machine learning analyses in this study demonstrated that one animal was central to the group with an influence on other group members. This is similar to other studies of cattle behaviour where certain individuals have social centrality and/or influence on the group’s behaviour [6,18]. An animal may have a strong influence as a result of age, dominance, or sex [6,52], but it is unknown why animal 6 in this study was an influential individual. Animals were of similar ages and animal 6 was not the only female in the mixed-sex group. Animals 1 and 7 were identified as more isolated from the rest of the group and they were also female, but the remaining females (n = 4) were similarly grouped with the steers. Animal 6 may have been the most dominant, but this would need to be confirmed across different scenarios. Further testing of temperament and dominance of all individuals may clarify reasons for certain individuals to be central versus excluded including subsequent potential impacts on their welfare such as affective state and stress reactivity [53]. The presence of both affiliative and avoidance (or repulsive) relationships are similar to results found in dairy cattle using data obtained from video observations, or proximity sensors [2,3,18]. Previous research has also demonstrated that not all individuals have strong social preferences [3,17]. Social relationships could develop through similar preferences for specific resources and areas [3,4] or observed social proximity greater than expected by chance could just be an artifact of similar resource use patterns such as preferred pasture grazing areas [4]. Boyland et al. [18] did confirm that social associations measured by proximity loggers were significantly correlated with the degree of allogrooming but not with observed agonistic interactions. The resolution and frequency of interactions obtained by the devices in the current study may be better able to detect subtle social effects thus improving the ability to quantify all types of social relationships.

The relationships in the current study were determined over a 25-min period. Previous research from video observations indicated social relationships were moderately to strongly stable across two observation periods six months apart [2], but proximity loggers showed dynamic social relationships in dairy cattle where group composition was continually changing [18]. Rocha et al. [17] monitored dairy cattle across a 17-week period using sensors and showed stability in social relationships across this period in a fixed group but the relationships depended on the area in which the contacts were recorded. The established networks and associations were also disrupted when group composition was changed through the introduction and removal of some individuals [17]. It is unknown if the relationships identified in this study would be repeatable across time and across different situations. The cattle in this study had been housed as a group of 12 individuals but only 10 devices were available for the study. The role of the other two individuals excluded from the assessment is unknown. If stable social relationships could be confirmed from one short sampling period, then this could dramatically reduce the direct animal handling and observation time needed to quantify differing social positions of specific individuals.

The results from one study period of a short duration indicated that social interactions could be quantified and animals of different social positions within the group identified. While the devices applied in this study are not yet practically feasible to apply longer-term, or in a commercial scenario, similar to other wireless tracking technologies [54], there is high value in continued research. Precise data on a finer-scale resolution than what GPS devices provide could be used in future studies to further understand the role of individuals within groups and why some animals may be central to the network versus repelled. Correlations between social interactions and multiple physiological and behavioural assessments could determine if more time spent alone is indicative of illness or differing personality traits, coping styles, or affective states. These repelled individuals could present higher stress levels and potentially lower growth or productivity. Removal of either central or repelled individuals could have contrasting impacts on the behaviour and welfare of the group. Additionally, further testing could confirm if individuals classified as central or repelled in one group maintain a similar position if moved to a new group of individuals.

The application of the findings from this study and demonstration of the technology and data analysis techniques was limited due to the use of one group for a relatively short period. The period of study (25 min) was restricted by the power requirements as the prototype devices require frequent sampling to ensure high accuracy and this drains the battery and limits the potential for long-term deployment. In addition, the robustness of the prototypes to withstand cattle damaging the casing from rubbing or social interaction are needed for longer-term application in a paddock situation. Another consideration is that no assessment of the impact of the attachment of the girth and devices was conducted and while cattle did not appear to be agitated or affected by the attachment of the devices, there is the possibility that social behaviour may have been impacted. With the development of a more robust weatherproof device with greater battery power, a widespread application could be achieved.

## 6. Conclusions

In this paper, based on the location information acquired by cooperative wireless localisation, machine learning algorithms were applied to analyse the social structure of a herd of cattle and the social behaviour of the individuals. These algorithms mined the location information related to social attraction and repulsion and were able to quantify the affinity of cattle for one another within a group. Social networks were present in the small herd of cattle, and the localisation devices provided more accurate information than would otherwise be obtained from just video recordings or live observations, such as animals with greater affinity and influence on others. Replication with the devices across more groups and repeatability within groups across different settings would confirm the value of quantifying social interactions with this degree of precision. Further work is needed to apply the devices for extended periods of time, as well as improve the robustness of the devices to be deployed on animals.

## Figures and Tables

**Table 1:** The closeness between every pair of animals in the experiment as characterised by $W_{i,j}$. Here $W_{i,j}$ are unitless values between 0 and 10. Animal *i* is closer to animal *j* if $W_{i,j}$ is larger.

$W_{i,j}$	Animal	1	2	3	4	5	6	7	8	9	10
Animal											
1		0	6.06	3.61	5.28	4.45	4.11	4.32	5.79	6.19	5.19
2		6.43	0	5.29	4.92	3.52	3.65	5.81	5.34	4.90	5.15
3		5.46	5.70	0	4.69	5.19	4.24	4.12	5.81	4.43	5.36
4		6.68	5.27	4.94	0	3.62	4.10	6.48	5.23	4.58	4.10
5		6.17	4.34	5.26	4.35	0	3.99	6.69	4.36	4.79	5.05
6		6.10	4.55	4.71	4.72	3.98	0	5.59	5.27	5.28	4.79
7		4.86	5.59	3.28	4.62	5.13	3.55	0	6.75	5.40	5.83
8		6.62	5.78	5.29	4.99	3.63	4.79	7.31	0	3.52	3.06
9		7.39	5.29	4.65	4.94	4.42	4.81	6.39	3.40	0	3.69
10		6.51	5.79	5.61	4.48	4.55	4.65	6.91	2.99	3.49	0

## Appendix A

This section describes the EKF-based sensor fusion algorithm use in Section 2.3, which combines GPS position measurements and relative range measurements for accurate animal tracking. The state of the EKF at each time step *k* is defined as the aggregated positions and velocities of all animals as follows

(A1) $$\begin{array}{c} x_{k}≜\left[\begin{array}{c} p_{k}^{1}\\ v_{k}^{1}\\ \vdots \\ p_{k}^{N}\\ v_{k}^{N} \end{array}\right], \end{array}$$

where $p_{k}^{i}$ and $v_{k}^{i}$ (*i* = 1,…,*N*) denote the *i*-th animal’s position and velocity at time step *k*, respectively; and *N* is the total number of animals. The transition model of the EKF is given by the following constant velocity model

(A2) $$\begin{array}{c} x_{k+1}\;=\;Ax_{k}+u_{k}. \end{array}$$

where A is an *N*-element block-diagonal matrix with each sub-matrix as given by

(A3) $$\begin{array}{c} A^{\prime }=\left[\begin{array}{cc} I_{2} & \Delta tI_{2}\\ 0_{2} & I_{2} \end{array}\right], \end{array}$$

with $I_{2}$ and $0_{2}$ denoting the two-dimensional identity matrix and zero matrix, respectively. Δt is the interval between time steps *k* and k+1. $u_{k}$ represents the random velocity change between time steps *k* and k+1, as given by

(A4) $$\begin{array}{c} u_{k}=\left[\begin{array}{c} 0_{2}\\ z_{k}^{1}\\ \vdots \\ 0_{2}\\ z_{k}^{N} \end{array}\right], \end{array}$$

where $z_{k}^{i}$ follows a zero-mean Gaussian distribution with covariance $\Sigma_{x}$.

The EKF is updated with GPS position measurements and relative range measurements, using the following (linearised) observation models, as given by

(A5) $$\begin{array}{cc} p_{k}^{\mathrm{GPS}}= & H^{\mathrm{GPS}}x_{k}+v_{k}^{\mathrm{GPS}}, \end{array}$$

(A6) $$\begin{array}{cc} d_{k}^{i,j}= & ∥p_{k,i}-{\hat{p}}_{k,j}∥+H^{i,j}\left(x_{k}-{\hat{x}}_{k}\right)+v_{k}^{i,j},\\ & i,j\in \{1,\ldots ,N\},i\neq j, \end{array}$$

where $p_{k}^{\mathrm{GPS}}$ aggregates the *x*- and *y*-coordinates of all animals measured by the GPS; $H^{\mathrm{GPS}}$ is an *N*-element block-diagonal matrix with each sub-matrix as given by

(A7) $$\begin{array}{c} H^{{\mathrm{GPS}}^{\prime }}=\left[I_{2},0_{2}\right], \end{array}$$

where $v_{k}^{\mathrm{GPS}}∼N{\left(0,\Sigma \right)}_{v}$ characterises the GPS positioning error; $d_{k}^{i,j}$ denotes the range measurement between the *i*-th animal and *j*-th animal; ${\hat{p}}_{k,i}$ and ${\hat{x}}_{k}$ denote the estimated position of the *i*-th animal and the estimated EKF state at time step *k*, respectively;

(A8) $$\begin{array}{c} H^{i,j}\;=\;e_{2i-1}^{2N}⊗\frac{{\left(p_{k,i}-{\hat{p}}_{k,j}\right)}^{T}}{∥p_{k,i}-{\hat{p}}_{k,j}∥}-e_{2j-1}^{2N}⊗\frac{{\left(p_{k,i}-{\hat{p}}_{k,j}\right)}^{T}}{∥p_{k,i}-{\hat{p}}_{k,j}∥}, \end{array}$$

where $e_{i}^{2N}$ denotes a 2N-dimensional unit row vector with the *i*-th element equal to 1, ⊗ denotes the Kronecker product; and $v_{k}^{i,j}∼N\left(0,\sigma_{k,i,j}^{2}\right)$ is the measurement error associated with $d_{k}^{i,j}$.
